# Implications of hepatitis C virus subtype 1a migration patterns for virus genetic sequencing policies in Italy

**DOI:** 10.1186/s12862-017-0913-3

**Published:** 2017-03-07

**Authors:** Lize Cuypers, Bram Vrancken, Lavinia Fabeni, Nadia Marascio, Valeria Cento, Velia Chiara Di Maio, Marianna Aragri, Andrea Clemencia Pineda-Peña, Yoeri Schrooten, Kristel Van Laethem, Daniel Balog, Alfredo Focà, Carlo Torti, Frederik Nevens, Carlo Federico Perno, Anne-Mieke Vandamme, Francesca Ceccherini-Silberstein

**Affiliations:** 1 KU Leuven – University of Leuven, Department of Microbiology and Immunology, Rega Institute for Medical Research, Clinical and Epidemiological Virology, Herestraat 49 – box 1040, 3000 Leuven, Belgium; 2 National Institute for Infectious Diseases L. Spallanzani-IRCCS, Rome, Italy; 3 Department of Health Sciences, Institute of Microbiology, School of Medicine, University of “Magna Graecia”, Viale Europa, Germaneto, 88100 Catanzaro, Italy; 4 Department of Experimental Medicine and Surgery, University of Rome Tor Vergata, Rome, Italy; 5 Global Health and Tropical Medicine, GHTM, Institute for Hygiene and Tropical Medicine, IHMT, University Nova de Lisboa, UNL, Rua da Junqueira 100, 1349-008 Lisbon, Portugal; 6 Molecular Biology and Immunology Department, Fundación Instituto de Immunología de Colombia (FIDIC), Basic Sciences Department, Universidad del Rosario, Bogotá, Colombia; 7 Luciad, Computer Software, Gaston Geenslaan 11, 3001 Heverlee, Belgium; 8 Department of Medical and Surgical Sciences, Unit of Infectious and Tropical Diseases, School of Medicine, University of “Magna Graecia”, Viale Europa, Germaneto, 88100 Catanzaro, Italy; 9 KU Leuven – University of Leuven, Department of Clinical and Experimental Medicine, Hepatology, Leuven, Belgium; 10 University Hospitals Leuven, Department of Gastroenterology and Hepatology, Leuven, Belgium

**Keywords:** HCV, HCV1a, Q80K, Italy, US, Europe, Phylogeography, Public health policy

## Abstract

**Background:**

In-depth phylogeographic analysis can reveal migration patterns relevant for public health planning. Here, as a model, we focused on the provenance, in the current Italian HCV subtype 1a epidemic, of the NS3 resistance-associated variant (RAV) Q80K, known to interfere with the action of NS3/4A protease inhibitor simeprevir. HCV1a migration patterns were analysed using Bayesian phylodynamic tools, capitalising on newly generated and publicly available time and geo-referenced NS3 encoding virus genetic sequence data.

**Results:**

Our results showed that both immigration and local circulation fuel the current Italian HCV1a epidemic. The United States and European continental lineages dominate import into Italy, with the latter taking the lead from the 1970s onwards. Since similar migration patterns were found for Q80K and other lineages, no clear differentiation of the risk for failing simeprevir can be made between patients based on their migration and travel history. Importantly, since HCV only occasionally recombines, these results are readily transferable to the genetic sequencing policy concerning NS5A RAVs.

**Conclusions:**

The patient migration and travel history cannot be used to target only part of the HCV1a infected population for drug resistance testing before start of antiviral therapy. Consequently, it may be cost-effective to expand genotyping efforts to all HCV1a infected patients eligible for simeprevir-based therapies.

**Electronic supplementary material:**

The online version of this article (doi:10.1186/s12862-017-0913-3) contains supplementary material, which is available to authorized users.

## Background

Hepatitis C virus (HCV) infected patients are nowadays treated with interferon-free regimens containing one or more antivirals that directly target virus proteins. These new direct-acting antivirals (DAAs) have a superb efficacy and, when combined, can clear the virus in more than 95% of the treated population, irrespective of the HCV genotype [1]. For most DAAs no clear association of particular naturally occurring resistance-associated variants (RAVs) with clinical outcome has been identified [2]. Because of this, baseline drug resistance testing is only recommended in well-defined cases such as the combination of NS3/4A protease inhibitor (PI) simeprevir with NS5B polymerase inhibitor sofosbuvir. This regimen is associated with reduced viral cure rates in HCV subtype 1a (HCV1a) cirrhotic patients who carry the naturally occurring, highly prevalent RAV Q80K [3–6]. The potential clinical relevance of the processes that shape the distribution of particular variants spurred an interest in the spatiotemporal aspects of the HCV1a evolutionary history in general, and in particular that of NS3 variant Q80K. Given the increased interest into the natural prevalence of several other RAVs, particularly located in NS5A [4, 7], insights into the patterns of HCV1a spread may be interesting for these variants as well.

Little over a decade after the discovery of the virus itself, it was revealed that there was a burst in the number of HCV1a transmissions starting from the second half of the 20^th^ century, which is likely linked to the history of illicit drug use and iatrogenic intervention rates [8–11]. While in these earlier studies the emphasis was on the temporal and spatial aspects of HCV1a in general, the medical relevance of the Q80K polymorphism drew attention to the segregation of the global HCV1a variability into two distinct clades, clade I and clade II [12–15], with the first clade further structured into three subclades [16]. In particular, Q80K strains are concentrated in the so-called clade IA [16], and the Q80K prevalence is largely accounted for by a single substitution event dating back to 1940–1963 [17]. This ancestral lineage first arose in the United States (US) and has been introduced multiple times into Europe [13, 14, 17]. Importantly, in these studies the migration patterns of HCV1a were evaluated on the continental geographical level, while a more fine-grained resolution is required to provide actionable information for national or regional public health programs.

Detailed phylogeographic analyses are particularly pertinent for clinically relevant lineages. Due to the rapid evolution of HCV treatments that achieve high viral cure rates, the regimen simeprevir with sofosbuvir, largely used as first-line treatment so far in Europe and in Italy [18, 19], has recently been categorized as suboptimal for HCV1a infected patients [20]. Nonetheless, second-line NS5A inhibitor-based regimens can still be considered when the absence of relevant RAVs is confirmed [21]. Despite this shift from NS3-targeting DAAs to those that inhibit the NS5A protein, a thorough analysis of the patterns of virus flow based on the NS3 region remains valuable because HCV only rarely recombines [22], and migration patterns inferred from small genomic regions hence have a genome-wide representativeness. Moreover, because drug resistance testing before treatment initiation has always been recommended for HCV1a infected patients when simeprevir is part of the planned regimen, NS3-based phylogeographic reconstructions can capitalize on a comparatively large number of publicly available NS3 sequence data to obtain the most accurate insights as possible. However, the rapid evolution in preferred HCV treatment schemes makes that phylogeographically informed public health decisions are of temporal value, and need to be updated regularly.

Here, we reconstructed the historical spread of HCV1a using a within-country-level resolution, and focused on the potential public health implications of the recovered clustering patterns for Italy. To this end, new sequencing data of the NS3 gene from Italy were combined with a selection of globally sampled publicly available sequences to infer the plausible origins and frequencies of HCV1a migrations into and out of Italy, and to explore the within-country migration flows for Italy, with statistical phylogeographic tools. In doing so, we provide a blueprint of how similar studies with a focus on other genomic regions in the HCV genome like the NS5A protein, or even other viruses can analyze patterns of virus flow by integrating genetic, spatial and temporal information in a state-of-the-art Bayesian phylogenetic framework. Furthermore, the timing of the Q80K origins was refined and the historical context of their spread from the US to Europe and Italy was discussed.

## Methods

### Newly generated Italian sequences

This retrospective study includes 183 samples, collected between 2011–2015, from mostly DAA-naïve (*N* = 163, 89.1%) Italian patients infected with HCV1a. The Italian strains were collected in at least seven regions (Additional file 1: Table S1). The NS3 protease gene (181 amino acids) was sequenced as reported elsewhere [23] in the context of routine clinical care at University of Rome Tor Vergata, Italy (art. 6 and art. 9, leg. 211/2003 and 196/2003).

### Sequence dataset compilation

The Italian sequence data were complemented with all publicly available HCV1a NS3 sequences from Genbank (*N* = 3032, http://www.ncbi.nlm.nih.gov/nucleotide) that fulfilled the following quality criteria. Only sequences that were unambiguously subtyped as HCV1a with subtyping tools Oxford HCV version 2 [24], COMET [25], and by phylogenetic analysis including a broad panel of reference sequences, were retained. The latter phylogenies were estimated on 1000 bootstrapped alignments with RAxML under a GTR + Γ substitution model, with subtypes assigned to strains clustering with ≥70% bootstrap support. Clonal sequences and strains from non-human hosts, duplicate sequences and strains covering <80% of the NS3 gene were removed. Remaining strains were aligned using an *in house* pairwise codon aware alignment tool (http://regatools.med.kuleuven.be/sequencetool/sequencetool.wt), followed by the removal of sequences with stop codons, leaving 930 taxa. Of these, 855 sequences with known sampling time and location were used for the phylogeographic reconstructions. A preliminary analysis revealed a large clade with only US-derived sequences that did not contain NS3 RAVs and that received 100% bootstrap support. As the within-US circulation was not the focus of this study, sequences from this clade were discarded to reduce computational burden in subsequent Bayesian analyses, resulting in a final set of 610 strains (Table 1).

**Table 1:** Overview of the complete dataset used in this study

Geographic origin	HCV1a dataset (n, % dataset)	Q80K (n, % country)	Sample time span
Italy^a^	280 (45.9)	52 (18.6)	2004-2015
US^b^	145 (23.8)	58 (24.6)^c^	1989-2008
Germany	67 (11.0)	19 (28.4)	2003-2013
Switzerland	46 (4.5)	6 (13.0)	2002-2006
Belgium	25 (4.1)	8 (32.0)	2007-2013
Thailand	19 (3.1)	3 (15.8)	2007-2010
France	13 (2.1)	/	2007-2011
Spain	5 (0.8)	/	2001-2002
UK	4 (0.7)	/	2008-2010
Brazil	2 (0.3)	/	2001-2003
Australia	2 (0.3)	/	2007
China	1 (0.2)	/	2009
Japan	1 (0.2)	/	2009
Total	610	146 (23.9)	1989-2015

The absolute and relative contribution of each country, the number of Q80K variants and their share by country, and the time span of the samples included per country, are detailed. ^a^183 (63%) of the Italian sequences have not been published before. ^b^To assess the proportion of Q80K strains, the 91 taxa from the removed cluster were taken into account (see Methods). ^c^Since the Q80K prevalence in the US was lower than expected, it was recalculated taking strains without sampling time information into account, resulting into a prevalence of 57.5%

To assess the robustness of the phylogeographic reconstructions against sampling biases, three random down-samples with a maximum of 30 sequences per location were created, resulting in three sets with a more balanced number of samples by location. Additionally, a diverse subset of 83 sequences was selected from clade I using the same procedure as in [26] to independently estimate the evolutionary rate in the Q80K clade. Briefly, from each sampling year the five most divergent sequences were selected with the Phylogenetic Diversity Algorithm [27], which selects the subtree of *n* taxa connected by the largest sum of branch lengths.

### Identification of Italian transmission networks

Transmission clusters that represent the Italian transmission dynamics were identified using a posterior root node support cutoff of ≥90% and were required to consist at least for 90% of member taxa isolated in Italy.

### Bayesian estimation of time-calibrated trees

All phylogenies were estimated using the Bayesian Evolutionary Analysis by Sampling Trees software (BEAST, v.1.8.2.) [28] with BEAGLE [29] to improve the computational performance. The skygrid or skyride model was used as a flexible non-parametric prior for the coalescent process in all analyses. A check for molecular clock signal [30] revealed that there was insufficient accumulation of divergence over the sampling time span to reliably estimate evolutionary rates. A first attempt to remedy this was specifying an informative prior distribution on the mean clock rate parameter of the relaxed clock model [31] that is based on a previously reported HCV1a dataset with strong temporal signal [32]. Exploratory runs with this model setup, however, indicated this still represented an overparameterization, which is why the mean clock rate was fixed to the mean of the NS3 rate estimate from the Gray et al. (2011) data, but while still estimating the variance of the evolutionary rate. A codon position partitioned HKY model was fitted, allowing for Γ-distributed among site rate heterogeneity at each position. Convergence and mixing properties of the Markov chain Monte Carlo (MCMC) chains were assessed with Tracer v1.6. Results of several MCMC chains were combined after removal of the burn-in. Maximum clade credibility (MCC) trees were summarized using TreeAnnotator included in the BEAST package. Trees were visualized with FigTree v 1.4.2 (http://tree.bio.ed.ac.uk/software/figtree/).

### Phylogeographic reconstructions

Source-sink relations were evaluated with a discrete diffusion model that allows for different rates depending on the direction of movement [33]. The migration pathways that are most relevant in the history of spread were identified with a Bayesian stochastic search variable selection (BSSVS) approach [34]. Bayes factor (BF) support (with BF ≥3 assumed to be relevant [35]) for all possible types of location exchanges was calculated using SpreaD3 [36]. Posterior expectations of the number of transition events between all possible pairs of locations (Markov jumps) were estimated with efficient stochastic mapping techniques [37].

## Results

### Sequence dataset

The final alignment included 610 sequences and covered 181 amino acids of the NS3 gene. Italy is the best-represented country in this final dataset, followed by the US (Table 1). Of the Western European strains, 36.4% was isolated in countries other than Italy. The Q80K variant was detected in 23.9% of all strains, and a somewhat lower prevalence of 19.3% was observed for Western Europe. The proportion of taxa carrying Q80K varied from 13.0% to 32.0% by country (Table 1).

### Multiple independent introductions of HCV1a and Q80K in Italy

Bayesian phylogeographic methods were used to determine when and from where HCV1a strains were introduced into Italy. As only limited sequence data was available for most European countries, all non-Italian isolates from Europe were grouped as ‘Europe (not Italy)’. Similarly, isolates sampled outside the US and Europe were grouped as ‘others’. To mitigate the potentially biasing effect of sample sizes on ancestral reconstructions of the location states, three subsampled datasets with more equal sample numbers by location state were analyzed (US, Italy, Europe (not Italy) and others).

**Fig. 1:**
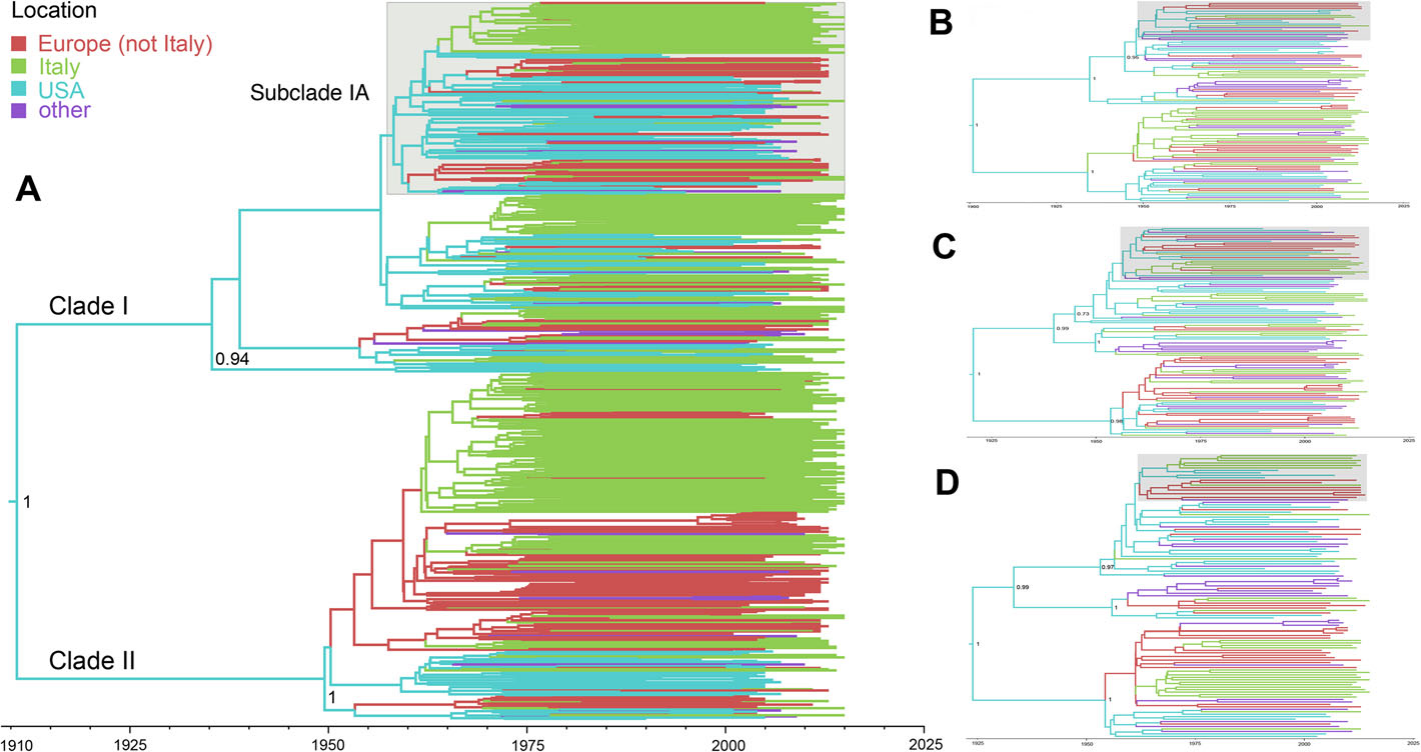
The United States as the immediate origin of HCV1a spread to Europe and Italy. The branches in the HCV1a maximum clade credibility (MCC) tree constructed from the complete dataset are colored according to sampling country with strains grouped into four locations (see legend). All but five Q80K strains are found in the same clade, which is highlighted in grey. We refer to Fig. 3 for further details on the Q80K history. Mixing of strains among the four locations can be observed in the entire tree. HCV entered Italy via the US and continental migration in the two major clades of HCV1a variability (Clade I and II) as well as in the Q80K clade. Posterior root node support is visualized in a selection of deeper nodes. **a**: entire dataset. **b**: MCC tree estimated from subsample 1 (see Methods). **c**: MCC tree estimated from subsample 2. **d**: MCC tree estimated from subsample 3

Independent of the sampling, similar migration patterns were observed in the two major HCV1a clades as well as among the clade 1 subclades, including the clade that comprises virtually all Q80K lineages, to which we from now on refer to as the Q80K clade (Fig. 1a-d). The most clear re-emerging pattern in the complete and in the subsampled datasets is the seeding, but not receiving, role of the US in the global spread of HCV1a. Specifically, the migration rates involving the US as origin location always receive high support for being non-zero, while rates of migration towards the US are indistinguishable from zero (Table 2). The remaining three well-supported migration links in the complete dataset received substantial Bayes factor (BF) support in two out of three subsampled datasets. Two of these links involve Italy, and lend support for Italy as a source and a sink for European continental virus movements. An interactive web-based visualization and demo video of the phylogeographic history of HCV1a in general and in particular of the Q80K polymorphism is available at http://demo.luciad.com/rega/?hepa&2d (legend, see Additional file 2).

**Table 2:** Posterior probabilities for all possible types of migrations

Migration from	Migration to	Complete dataset	subsample 1	subsample 2	subsample 3
Europe (not Italy)	Italy	**29.12**	2.88	**8.48**	**19.43**
	US	0.01	0.08	0.09	0.08
	Other	**7.66**	0.81	**5.88**	**11.07**
Italy	Europe (not Italy)	**10.28**	**18.23**	**4.37**	0.52
	US	0	0.06	0.07	0.05
	Other	0.07	**8.64**	0.47	1.15
US	Europe (not Italy)	**21.68**	**19.08**	**23.34**	**21.42**
	Italy	**22.37**	**22.88**	**24.37**	**12.93**
	Other	**8.74**	**25.61**	**31.01**	**29.45**
Other (not Europe –not US)	Europe (not Italy)	0.03	1.3	0.37	**3.61**
	Italy	0.03	0.33	1.43	0.23
	US	0	0.11	0.11	0.08

Posterior probabilities for migration rates supported by a BF ≥3 are marked in bold, indicated for the complete dataset as well as for the three subsamples created as described in methods



**Additional file 2: Visualization.** An interactive web-based visualization of the phylogeographic history of HCV1a. Migration patterns of HCV1a in general and in particular of the Q80K polymorphism are visualized in an interactive web application, available at http://demo.luciad.com/rega/?hepa&2d. A demo-movie with voice-over is accessible through a button implemented in the application, explaining the possibilities of the tool. More specifically, the world map is visualized in the upper panel, with the countries sampled in this study highlighted in a color and labeled by their name. The map shows how viral lineages are introduced/exported in/to different countries throughout time, represented by jumping lines on the upper panel. Viral lineages that circulate locally are represented by circles on the world map, with their size proportional to the number of branches. The visualization is simulated over time, using a fixed window of time as a filter. The length of a jump on the map, is inversely proportionate to the length of the branch in the phylogenetic tree. That is, longer geographic jumps on the map indicate branches that quickly traveled over large distances, while short jumps indicate branches that slowly traveled over relatively small distances. The timing of these jumps is indicated in the lower panel, which represents a timeline that plots new viral lineages over time. The colors in this timeline match the region of the same color on the geographic map. On the map, a darker color, indicates a larger viral lineage amount. Using the tool, we can apply a color filter on the branches of the tree, visualizing not only the phylogeographic history of HCV1a in general, but also of variant Q80K. Migration jumps will be colored by amino acid polymorphisms present at position 80 of the NS3 protein region, with amino acid Q indicated in red, and variant K in yellow. All other polymorphisms (being not Q or K) are colored blue, as indicated in the legend. (MP4 162 mb)


In addition to the significance of migration rates, the expected proportion of all possible migration events in the HCV1a history (Markov jumps) was also inferred (Table 2). Little over half of all expected migrations are virus movements from the US, and a majority of these are directed towards Western Europe. The virus flows involving Italy account for 83.4% of the remaining expected number of jumps, and Italy mostly acts as the destination location in this European continental spread (73.9%). Using the entire dataset, the first introduction of HCV1a into Italy was timed around 1957.7 (95% HPD: 1949.0-1964.4) (Fig. 1), and the first appearance of a Q80K lineage in Italy around 1961.5 (95% HPD: 1957.6-1965.7).

**Fig. 2:**
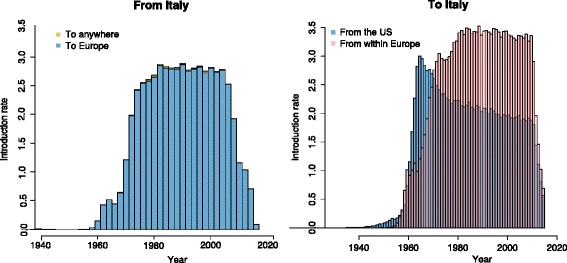
Immigration and emigration rates of HCV1a strains from and to Italy, over time period 1940–2015. *Left panel*: Over 99% of exportation events from Italy are directed towards other European countries (in *blue*). Note that the rate decline in the most recent years reflects the loss of branches in the sampling time period, rather than the start of an actual tendency. *Right panel*: The origin of virtually all strains entering Italy can be traced back to the United States (US) and other European countries (see Table 2). At the onset of the Italian HCV1a epidemic, immigration was most intense from the US (in *blue*). From 1970 onwards, introductions from the European continent started to dominate (in *red*). The introduction rate (y-axis) was defined as the median posterior estimate of the expected number of migration events from that particular location per time unit

The picture painted by the Markov jumps analysis above is a static one and does not inform us on changes over time of the relative importance of the migration flows to and from Italy. A plot of the expected number of migration events from Italy reveals a stable pattern of seeding almost uniquely towards other Western European countries throughout the history (Fig. 2, left panel). In contrast, a similar plot for the incoming migrations shows that the initial dominance of the US as origin location has decreased in favor of European continental HCV1a circulation, starting from 1970 (Fig. 2, right panel).

### Migration patterns within Italy

Twelve highly supported small clusters with only Italian isolates (size range: 2–7) and one pure Italian large cluster (size 34, cluster 13 in Table 3) were identified in the MCC summary tree of the time evolutionary histories (Additional file 3: Figure S1). Information on the sampling region (North, Central, South) was available only for a limited number of the members of the smaller clusters and for the majority of samples from the large cluster (Table 3). Both homogenous (*n* = 1, cluster 4) and mixed-origin (*n* = 1, cluster 2) small clusters were observed. Phylogeographic analysis of cluster 13 with the same (asymmetric) model as before painted a picture of migration by proximity where both North and South regions interact with the Central region (BF support >3) and vice versa (BF support >20), but not with each other (BF support for North-to-South and South-to-North migration rates <3). There is one larger Italian cluster in the Q80K clade (Fig. 1), but this has a posterior root node support of 81%, which is below the used cut-off. Nonetheless, the distribution of its lineages over at least five regions (Additional file 4: Table S2) also points to an interregional mixing of strains within Italy.

**Table 3:** Highly supported Italian clusters identified in the MCC tree obtained from the complete dataset

	Sample size (number of strains)	Tree root support (posterior probability)	Presence of Q80K (N, %)	Time span (years)	Regional information
Cluster 1	2	1	0 (0)	2.23	Unknown: 2
Cluster 2	2	1	0 (0)	3.4	North: 1 – South: 1
Cluster 3	2	1	2 (100)	8.4	Unknown: 2
Cluster 4	2	1	2 (100)	15.0	Central: 2
Cluster 5	2	1	0 (0)	17.6	Unknown: 2
Cluster 6	2	0.98	2 (100)	22.8	Unknown: 2
Cluster 7	2	0.99	0 (0)	32.3	Unknown: 2
Cluster 8	2	0.97	0 (0)	32.8	Central: 1 – Unknown: 1
Cluster 9	2	0.92	0 (0)	33.1	Unknown: 2
Cluster 10	5	0.99	0 (0)	13.9	Unknown: 5
Cluster 11	6	1	0 (0)	12.6	Central: 1 – Unknown: 5
Cluster 12	7	1	0 (0)	7.8	Central: 6 – Unknown: 1
Cluster 13	34	0.98	3 (8.8)	35.4	North: 2 - Central: 21 - South: 7 – Unknown: 4

In total, 13 highly supported clusters were identified (posterior root node support ≥0.90), with 2 to 34 Italian member taxa. Sampling region information was unavailable for many taxa. For each cluster, the sample size, posterior root node support, presence of Q80K and the time spanned by the cluster, is listed. Clusters are ordered according to sample size and time span

### Refining Q80K divergence times

**Fig. 3:**
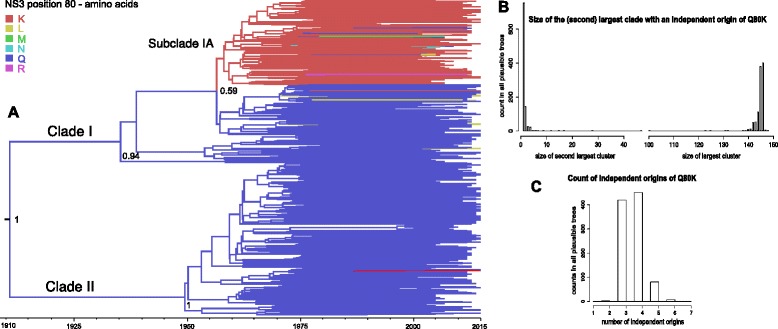
**a**: A founder effect dominates the history of the Q80K clade. The branches in the HCV1a MCC tree are colored according to the inferred amino acid at NS3 position 80 (see legend). All except five Q80K variants descend from a common ancestor that is inferred to have existed around 1957 (95% HPD: 1947–1959). Posterior root node support is visualized in a selection of deeper nodes. Amino acids other than Q or K were found in only 13 lineages. **b**: Histogram of the size of the (second) largest clade with an independent origin of Q80K among the plausible trees. The size of the largest clade remains more or less constant, and the limited size of the second largest clade shows that most contemporaneous Q80K lineages reside in the major Q80K clade. **c**: Histogram of the number of independent origins of a Q80K lineage in the plausible trees. This shows that in the most plausible trees three to four *de novo* appearances of the Q80K variant appear

As investigating the virus migration patterns entails estimating time-calibrated histories, we seized the opportunity to briefly re-evaluate the temporal aspects of the Q80K origins, but now taking among lineage rate variation explicitly into account. The Q80K variant has evolved independently on multiple occasions, and one lineage was particularly successful and gave rise to almost all currently circulating Q80K strains. This major Q80K clade had a low posterior support (58.6%), but upon further inspection it became clear that this was due to one or a few taxa that frequently clustered outside this dominant Q80K clade (see Fig. 3b-c). The genesis of the most recent common ancestor of the most successful Q80K clade was estimated around 1957 (95% highest posterior density (HPD): 1947.3-1958.6).

In this analysis the mean clock rate was fixed to the mean of the NS3 rate estimate obtained from the Gray et al. (2011) HCV1a full-genome data [32]. To verify whether this did not bias our timing of the origin of the Q80K clade, a ‘Q80K clade rate’ was estimated independently using a subset of this clade’s taxa selected in the same way as in [26] (see Methods). Reassuringly, the NS3 mean rate estimates in both analyses were nearly identical to each other (1.02x10^−3^ substitutions/site/year for the Q80K clade, and 1.00x10^−3^ for the HCV1a NS3 gene using the dataset of [32]), showing that the latter was an appropriate external calibration.

## Discussion

In this work, we elucidated patterns in the HCV1a transmission dynamics that are relevant for the Italian HCV sequencing policy, with a focus on the NS3 variant Q80K, which can affect success of simeprevir containing drug regimens and serves as a model for other clinically relevant RAVs. To this end, newly obtained Italian NS3 sequences were combined with all publicly available sequences worldwide obtained from isolates with known sampling time and location. Sequence evolution was integrated with spatial diffusion at a country-level resolution to assess the relative importance of local spread versus virus importation into Italy. The within-Italy migration dynamics were tentatively explored and the opportunity was taken to concisely revisit the dating of the highly successful Q80K founder event.

The clustering patterns of Italian strains with globally sampled ones showed that HCV1a, including Q80K strains, entered Italy on multiple occasions. The origin of these lineages can be traced to both the US and Western Europe, and no significant link with other locations was found (Table 2). Few samples were available from other regions with a considerable HCV1a burden, such as the Andean Latin American and Caribbean regions (Table 1, [38]). While this may have affected the inferred role of the US as the origin (but see [8]), it does not affect our findings that HCV1a, including Q80K strains, has been introduced into Italy on multiple occasions.

The plot of the expected number of migration events per year from the dominant origin locations (e.g. the US and Western Europe) to Italy shows that in the hypothetical situation of a 100% effective screening and treatment of immigrants and short-term travelers of these regions, approximately five to six lineages can be prevented from initiating an ‘Italian’ transmission network each year. Note that, because the sampling density impacts the extent of clustering, this estimate is a lower bound. Temporally resolving the seeding intensity by origin region also revealed a switch in the relative importance between both locations, initially mainly from the US, with European continental spread dominating from the late 1960s, early 1970s onwards (Fig. 2a, left panel).

Importantly, the Italian lineages are dispersed among all clade 1 subclades and there are no clear differences in migration pathways between clade I, which encompasses all but one of the Q80K strains, and clade II (Fig. 1). This has several potential public health policy implications. Because both virus import and local spread shape the Italian HCV1a, and thus also the Italian Q80K epidemic, immigrants and short-term travelers or other (non-travelling) Italian citizens cannot be specifically targeted or disregarded in a sequencing program. Moreover, since the origins of imported lineages in the Q80K and other clades are shared, and the Italian epidemic is usually seeded from those locations that contribute most to the Q80K clade, also referred to as clade 1A [16], this too cannot be used to distinguish populations in their relative risk of failing with simeprevir.

The national patterns of virus movements were explored by coarsely dividing Italy in a North, Central and South region, not only because detailed geographical data are only available for a subset of the Italian samples (see Additional file 1: Table S1) but also because using a higher-level resolution for this small dataset precludes well-informed parameter estimations. Although it remains difficult to extract general migration patterns from a limited sampling, the extensive mixing of lineages among different regions identified for both wild-type and Q80K lineages shows that the Italian epidemic likely is quite uniform and does not evolve in well-segregated local sub-epidemics. Of note, because of this it is likely that many unsampled Italian HCV1a lineages are part of already detected Italian transmission networks and thus represent the same introduction events (and origins) as those of sampled lineages.

HCV is characterised by substantial amounts of among lineage rate variation [32]. This cannot only interfere with divergence time estimation [39, 40] but also with phylogenetic inference [41, 42], and is to the best of our knowledge for the first time explicitly modelled in our timing of the Q80K origins. Moreover, unlike in earlier work, the phylogeny and divergence times were co-estimated using an integrated Bayesian approach that appropriately takes the shared ancestry into account and also avoids the risk of error propagation typical for rate-smoothing procedures that rely on a pre-specified topology. Our dating places the origin of the most successful lineage in 1955 (95% HPD: 1947.3-1958.7). This is far more recent than the penalised likelihood based point estimate by McCloskey et al. [17] and demarcates a more narrow interval than their linear regression based estimate (1955, 95% CI: 1945–1963). This makes that the earliest Q80K origin no longer overlaps with the dates of World War II (WOII) and, in turn, renders the hypothesis that the Q80K lineage was introduced into Europe through large-scale movements of military troops unlikely. Rather, this successful Q80K lineage found itself at the right time at the right place to profit from the post-WOII increase in parenteral iatrogenic procedures and illicit drug use, both in the US and elsewhere, including Italy [43, 44]. The majority of Italian Q80K strains for which sampling region information was available (156/183) were isolated in Central to Southern and Insular areas, the regions from where most Italy-to-US emigrants originate [45]. As many of these emigrants eventually returned - estimates range from 11% to 73% for the first half of the 20^th^ century [46, 47] - the introduction of HCV1a via remigration is an alternative hypothesis that deserves further exploration. Other Western European countries, in particular Germany and the UK, also have a history of intense travel, migration and trade with the US, but large discrepancies in the reported numbers of immigrants arriving and leaving the US hamper a comparison of the migration flow intensities (statistics US Homeland Security). In this respect it is noteworthy that the group of non-Italian European samples includes a reasonable 67 German samples but only four UK isolates (Table 1). The inclusion of additional data, in particular from the UK, can therefore be expected to ‘break’ some of the direct links between the US and Italy. Additionally, Latin America and South-East Asia, for which high HCV1a prevalence rates have been reported [38], were underrepresented in our dataset. This implies that a number of introductions into Western Europe (or Italy) from these locations likely were not detected. However, because Brockmann et al. [48] reported less intensive migration links between Europe and Latin America as compared to between Europe and North America, and virus dispersal is usually linked to the degree of human connectivity [49], we believe this did not heavily impact our results.

The rate at which new virus genetic data is generated is increasing as a result of upscaled sequencing efforts in the context of DAA combination therapies. These are frequently extensively annotated with socio-demographic and clinical metadata, which can be used for a fine-grained identification of processes that fuel an epidemic (see [50] for a recent example for HCV). Unfortunately, such metadata are usually not deposited along with the virus genetic data in public databases for reasons of privacy. Here, it has been highlighted how scrutinizing the population level transmission dynamics can still reveal information relevant for intervention strategies provided basic annotations such as sampling time and space are available.

## Conclusions

In-depth phylogeographical analyses revealed that the Italian HCV1a epidemic is shaped by complex patterns of virus importation and sustained local and interregional transmission. Because this precludes a clear differentiation of Q80K presence/absence between sub-populations, it may be cost-effective to test all HCV1a patients eligible for therapy with simeprevir for the absence of this polymorphism. Furthermore, this study demonstrates the usefulness of a versatile analysis strategy that can readily be applied in different regions or countries, to other genetic regions in the HCV genome such as the NS5A protein and to other measurably evolving pathogens.

## Additional files

Additional file 1: Table S1. The country-wide distribution of Italian samples. Overall, 183 sequences were newly obtained from seven different regions. The publicly available data trace back to the work by de Luca *et al*. [14] (*n* = 67) and Paolluci *et al*. [51] (*n* = 30). The former samples have an unknown distribution among the Lazio, Lombardy, Tuscany and Apulia region. For the latter, it is only known that the patients visited the Fondazione IRCCS Policlinico San Matteo, Pavia, Lombardy. (DOC 31 kb)
Additional file 3: Figure S1. The thirteen highly supported Italian clusters indicated in the maximum clade credibility summary tree. The highly supported Italian-only clusters are highlighted in green, and the cluster number corresponding to the numbering in Table 3 is also detailed. The red background marks the dominant Q80K clade. The evolutionary distance bar at the bottom indicates the percentage of nucleotide substitutions per site along each lineage. (PDF 50 kb)
Additional file 4: Table S2. Geographical distribution of Italian Q80K lineages. For each Italian strain within the large Q80K clade, more detailed regional information is listed. (DOC 29 kb)

## Abbreviations

BEAGLE: Broad-platform Evolutionary Analysis General Likelihood Evaluator
BEAST: Bayesian Evolutionary Analysis Sampling Trees
BF: Bayes Factor
BSSVS: Bayesian stochastic search variable selection
CI: Confidence interval
DAA: Direct-acting antiviral
GTR: Generalized time reversible model
HCV: Hepatitis C virus
HIV: Human immunodeficiency virus
HKY: Hasegawa-Kishino-Yano
HPD: Highest posterior density interval
MCC: Maximum clade credibility
MCMC: Markov Chain Monte Carlo
n: Number
PI: Protease inhibitor
RAV: Resistance-associated variant
RAxML: Randomized Axelerated Maximum-Likelihood
US: United States
WOII: World War II
