# Supplementary material for: Implications of hepatitis C virus subtype 1a migration patterns for virus genetic sequencing policies in Italy
Source: BMC Evol Biol. 2017 Mar 7;17:70. doi: 10.1186/s12862-017-0913-3 (PMC5341469; doi:10.1186/s12862-017-0913-3)
Supplement: Additional file 1: Table S1. — The country-wide distribution of Italian samples. Overall, 183 sequences were newly obtained from seven different regions. The publicly available data trace back to the work by de Luca et al. [14] (n = 67) and Paolluci et al. [51] (n = 30). The former samples have an unknown distribution among the Lazio, Lombardy, Tuscany and Apulia region. For the latter, it is only known that the patients visited the Fondazione IRCCS Policlinico San Matteo, Pavia, Lombardy. (DOC 31 kb) [file 12862_2017_913_MOESM1_ESM.doc]

**Table S1: The country-wide distribution of Italian samples.**

Overall, 183 sequences were newly obtained from seven different regions. The publicly available data trace back to the work by de Luca *et al*. [14] (n=67) and Paolluci *et al*. [51] (n=30). The former samples have an unknown distribution among the Lazio, Lombardy, Toscane and Puglia region. For the latter, it is only known that the patients visited the Fondazione IRCCS Policlinico San Matteo, Pavia, Lombardy.

| **Geographical region (%*)** | **Geographical region** | **Number of taxa (n,%)** |
| --- | --- | --- |
| Northern Italy (26.0) | Lombardy | 10 (3.6) |
|  | Liguria | 14 (5.0) |
|  | Emilia Romagna | 2 (0.7) |
| Central Italy (61.6) | Lazio | 101 (36.1) |
|  | Abruzzo | 38 (13.6) |
| Southern Italy (12.4) | Sardinia | 17 (6.1) |
|  | Puglia | 1 (0.4) |
| Unclear | Unclear | 97 (34.6) |

* Fractions are estimated assuming that the samples obtained by de Luca *et al.* [14] are equally distributed among the possible regions (with Lazio and Toscane being part of Central Italy – Lombardy of North Italy and Puglia of South Italy), and that all samples by Paolluci *et al*. [51] are from Lombardy.
