# Supplementary figures and images for: Implications of hepatitis C virus subtype 1a migration patterns for virus genetic sequencing policies in Italy
Source: BMC Evol Biol. 2017 Mar 7;17:70. doi: 10.1186/s12862-017-0913-3 (PMC5341469; doi:10.1186/s12862-017-0913-3)

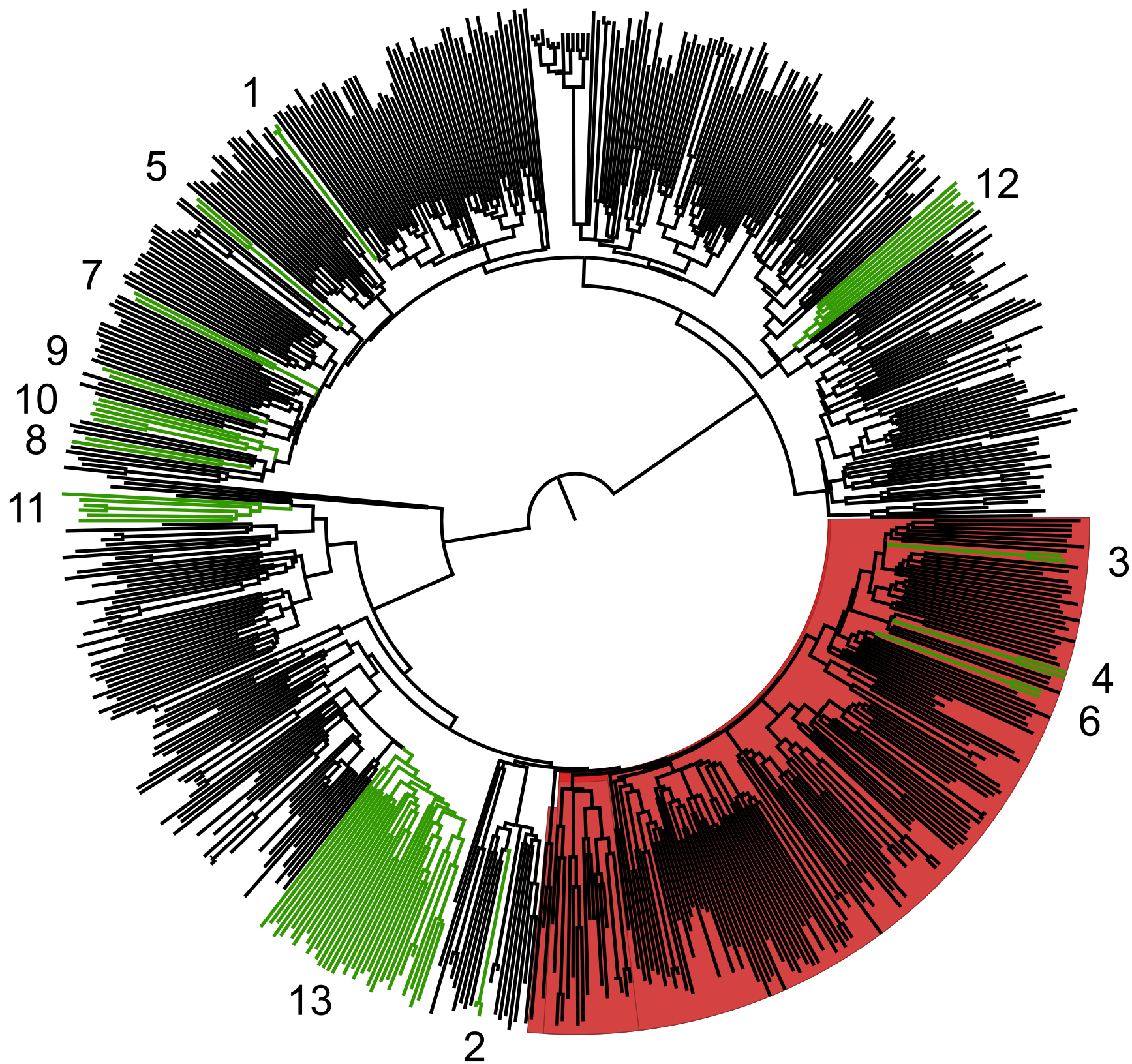

20.0

Supplement: Additional file 3: Figure S1. — The thirteen highly supported Italian clusters indicated in the maximum clade credibility summary tree. The highly supported Italian-only clusters are highlighted in green, and the cluster number corresponding to the numbering in Table 3 is also detailed. The red background marks the dominant Q80K clade. The evolutionary distance bar at the bottom indicates the percentage of nucleotide substitutions per site along each lineage. (PDF 50 kb) [file 12862_2017_913_MOESM3_ESM.pdf]
