# Supplementary material for: Implications of hepatitis C virus subtype 1a migration patterns for virus genetic sequencing policies in Italy
Source: BMC Evol Biol. 2017 Mar 7;17:70. doi: 10.1186/s12862-017-0913-3 (PMC5341469; doi:10.1186/s12862-017-0913-3)
Supplement: Additional file 4: Table S2. — Geographical distribution of Italian Q80K lineages. For each Italian strain within the large Q80K clade, more detailed regional information is listed. (DOC 29 kb) [file 12862_2017_913_MOESM4_ESM.doc]

**Table S2: Geographical distribution of Italian Q80K lineages.** For each Italian strain within the large Q80K clade, more detailed regional information is listed.

| **Geographical region** | **Geographical province** | **Number of taxa (n,%)** |
| --- | --- | --- |
| Northern Italy | Lombardia | 1 (2.4) |
|  | Liguria | 1 (2.4) |
| Central Italy | Lazio | 7 (17.1) |
|  | Abruzzo | 4 (9.8) |
| Southern Italy | Sardinia | 6 (14.6) |
| Unknown [14,52] | Unknown | 22 (53.7)* |

* [14]: 20 taxa with unknown distribution among regions Lazio, Lombardy, Toscane and Puglia
 [52]: 2 taxa most probably from Lombardy
